# Errors in Abstract, Results, and Tables

**DOI:** 10.1001/jamanetworkopen.2025.30317

**Published:** 2025-08-11

In the Original Investigation titled “Utility of the US Preventive Services Task Force for Preeclampsia Risk Assessment and Aspirin Prophylaxis,”^1^ published July 17, 2025, there were errors in the Abstract, Results, and Tables. In the results section of the Abstract and the fourth paragraph of the Results, it was reported that obesity was not associated with preeclampsia, when there was a small-magnitude association (risk ratio, 1.11; 95% CI, 1.01-1.22; *P* = .048). In the second paragraph of the Results section, the denominator of a percentage was misreported as, “Of 5046 participants, 5046 (88%) were at increased (moderate or high risk) of preeclampsia.” This should be “Of 5684 participants, 5046 (88%) were at increased (moderate or high risk) of preeclampsia.” In Table 1, the footnote defined the moderate risk category as “including those with a BMI of 30 or greater, advanced maternal age, Black race, and or nulliparity,” which should have been labeled as the moderate 1+ risk category; the same correction was made in the footnote for Table 3. In the fourth paragraph of the Results, the sentence, “AMA [advanced maternal age], Black race, and obesity were each significantly associated with the high risk category” was updated to read “within the high risk category.” In the fifth paragraph of the results it was reported that “among participants with obesity (BMI [body mass index] ≥30), a higher proportion were recommended AP [aspirin prophylaxis].” This should have stated that a lower proportion were recommended AP. In Table 2, the 95% CI for BMI (≥30) was misreported as 1.01 to 1.23; this was corrected to read 1.01 to 1.22. The footnote in Table 2 was updated to provide clarity on the definition of the moderate 1 risk category. Finally, in the first column of Table 3, the moderate 1+ risk category was incorrectly labeled as moderate. This article has been corrected.^1^
